# Methionine residues around phosphorylation sites are preferentially oxidized *in vivo* under stress conditions

**DOI:** 10.1038/srep40403

**Published:** 2017-01-12

**Authors:** Francisco J. Veredas, Francisco R. Cantón, J. Carlos Aledo

**Affiliations:** 1 Departamento de Lenguajes y Ciencias de la Computación, Universidad de Málaga, 29071-Málaga, Spain; 2 Departamento de Biología Molecular y Bioquímica, Facultad de Ciencias, Universidad de Málaga, 29071-Málaga, Spain

## Abstract

Protein phosphorylation is one of the most prevalent and well-understood protein modifications. Oxidation of protein-bound methionine, which has been traditionally perceived as an inevitable damage derived from oxidative stress, is now emerging as another modification capable of regulating protein activity during stress conditions. However, the mechanism coupling oxidative signals to changes in protein function remains unknown. An appealing hypothesis is that methionine oxidation might serve as a rheostat to control phosphorylation. To investigate this potential crosstalk between phosphorylation and methionine oxidation, we have addressed the co-occurrence of these two types of modifications within the human proteome. Here, we show that nearly all (98%) proteins containing oxidized methionine were also phosphoproteins. Furthermore, phosphorylation sites were much closer to oxidized methionines when compared to non-oxidized methionines. This proximity between modification sites cannot be accounted for by their co-localization within unstructured clusters because it was faithfully reproduced in a smaller sample of structured proteins. We also provide evidence that the oxidation of methionine located within phosphorylation motifs is a highly selective process among stress-related proteins, which supports the hypothesis of crosstalk between methionine oxidation and phosphorylation as part of the cellular defence against oxidative stress.

Reactive oxygen species (ROS) are best known as damaging agents linked to aerobic metabolism^1^. However, a more nuanced view has recently emerged. It is now clear that some ROS, such as hydrogen peroxide (H_2_O_2_), can act as messengers both in plants and animals^2^,^3^. Many of the cellular responses triggered by oxidative stress are known to be mediated by signalling cascades involving protein phosphorylation^4^,^5^. However, how H_2_O_2_ is detected and translated into a biological response is largely unknown. A direct means by which oxidants may be sensed and transduced into biological responses involves the reversible oxidation of amino acid side chains in cellular proteins. Sites that often undergo post-translational modification (PTM) have a functional group that can serve as a nucleophile in the modification reaction. In this regard, cysteine and methionine, the two sulfur-containing amino acids in proteins, are likely to be oxidized by mild oxidants^6^,^7^. While cysteine forms cystine through a disulfide linkage, methionine is oxidized to methionine sulfoxide (MetO) by addition of oxygen to its sulfur atom. Both oxidation reactions can be reversed by enzyme-catalyzed reactions. Disulfides are reduced back to their thiol form by various reductases^8^. On the other hand, MetO is reduced back to methionine by the methionine sulfoxide reductases, enzymes that are virtually present in all aerobic cells^9^. Whereas the roles of protein-bound cysteinyl residues as cellular redox regulators are well appreciated, those of methionine remain largely unexplored^6^.

Like phosphorylation, methionine oxidation is a reversible covalent modification. The mere addition of an oxygen atom to the sulfur atom of methionine, a reaction that may be enzyme-catalyzed^10^,^11^, can cause changes in the physicochemical properties of the whole protein, which, in turn, can affect the stability and/or the activity of the oxidized protein^2^,^12^. Thus, methionine oxidation has been demonstrated to both up-regulate^13^,^14^ and down-regulate^15^,^16^ protein activity through direct sulfoxidation of specific methionine residues. Moreover, methionine oxidation can also impact protein function indirectly by coupling oxidative signals to protein phosphorylation-dephosphorylation. In this regard, the activities of calcineurin, a protein phosphatase, as well as Ca^2+^/calmodulin-dependent protein kinase II, have been previously shown to be modulated by specific methionine oxidation^17^,^18^. Furthermore, it has been reported that the oxidation of methionine residues within the phosphorylation motif of nitrate reductase^19^ and pyruvate dehydrogenase^20^ inhibits the phosphorylation of nearby sites, providing a mechanism to couple oxidative signals to changes in protein phosphorylation. These isolated observations imply that reversible methionine oxidation might serve as a rheostat to control the phosphorylation of proximal phospho-acceptors^21^. Indeed, if this postulated crosstalk between O-phosphorylation and methionine sulfoxidation occurs broadly, it may have widespread implications for our understanding of redox signalling. However, the identification of PTM crosstalk at the proteome level remains a great challenge. As a first approach, Rao and co-workers performed computational analyses of the phosphoproteome of several species, concluding that the large proportion of known phosphorylation sites with methionine in their proximity fulfils the necessary condition for crosstalk^22^. On the other hand, the awareness that methionine oxidation may provide a mechanism for the redox-dependent modulation of a wide range of protein functions and cellular processes has prompted proteome-wide studies of methionine oxidation, which have allowed the identification of a large number of human cellular proteins as potential targets of oxidative signals^23^. In the current study, we have combined these data with those derived from the human phosphoproteome to address the occurrence of crosstalk between sulfoxidation and phosphorylation at a proteomic level. Our results suggest that there exists a large subset of methionine residues from the whole proteome that may be signalling-competent. In addition, we identify candidate proteins that may exhibit crosstalk between these two PTMs.

## Results

### Sulfoxidation coupled to other PTMs

**Figure 1:**
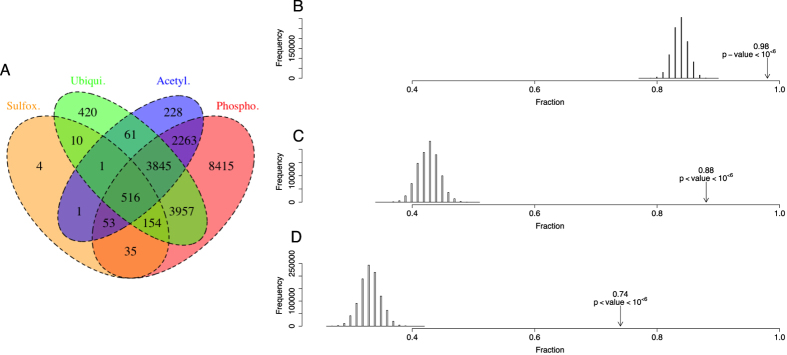
Association of methionine sulfoxidation with other post-translational modifications. (**A**) A Venn diagram showing the overlap between the human MetO-modified proteome with the phosphoproteome, the ubiquitinome and the acetyl-proteome. The highest overlap was observed with the phosphoproteome (98%), followed by the ubiquitinome (88%) and the acetyl-proteome (74%). More than half of the sulfoxidized proteins (approximately 67%) are simultaneous targets for all of the other three PTMs. To assess the statistical significance of these observations, 10^6^ samples of the same size as the MetO-proteome were randomly sampled from the whole human proteome and used to compute the null distribution (bar graphs) for the proportions of phosphorylatable (**B**), ubiquitinatable (**C**) and acetylable proteins (**D**).

Multiple PTMs within a protein can co-ordinately determine a functional outcome, thereby providing an expanded variety of mechanisms to integrate external and internal cues. While the regulatory importance of protein PTM has long been known, the coexistence of different PTMs on the same protein has only recently gained attention^24^,^25^,^26^,^27^. However, none of these recent studies have addressed the co-occurrence of methionine sulfoxidation and O-phosphorylation or other modifications. Thus, we addressed the co-occurrence of these PTMs within the same protein. We observed a surprisingly high overlap between proteins containing MetO and those exhibiting PTMs such as phosphorylation, ubiquitination and acetylation (Fig. 1A). We found that 98, 88 and 74% of sulfoxidized proteins are also phosphorylated, ubiquitinated and acetylated, respectively. To evaluate the statistical significance of these observations, we empirically generated null distributions by sampling the human proteome as described in the methodological section (Fig. 1B–D). The results of these analyses indicated that proteins susceptible to sulfoxidation are more likely to have a second PTM, when compared to other proteins that are not sulfoxidized (p-values < 10^−6^).

**Figure 2:**
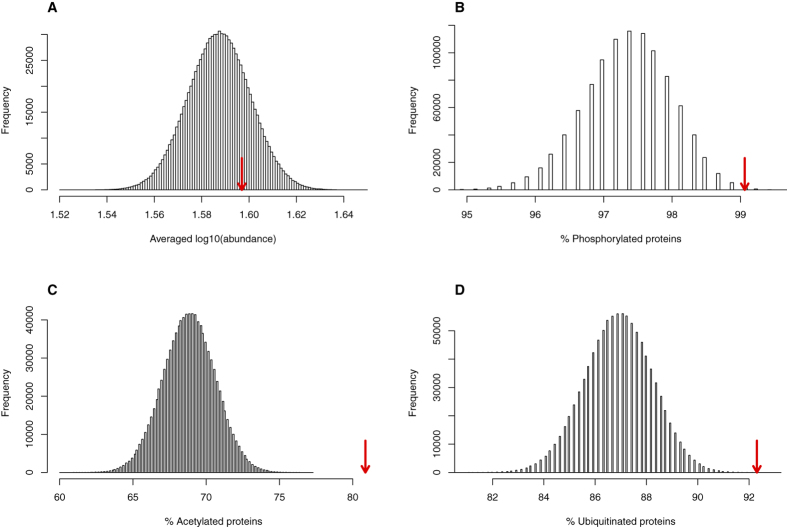
The high overlap between sulfoxidation and other PTMs remains after correcting for protein abundance. To generate empirical null distributions corrected for protein abundance, the Jurkat cell proteome was sampled to obtain 10^6^ samples of the same size (533 proteins) as the MetO-proteome (proteins containing MetO for which their abundances are known). The sampling was random but was constrained to provide samples with protein abundances comparable to those observed for sulfoxidized proteins. These samples were used to construct the null distribution of the averaged decimal logarithm of the abundance (**A**), and the percentage of phosphorylated (**B**), acetylated (**C**) and ubiquitinated (**D**) proteins. The values of these variables computed for the collection of proteins are referred to as the MetO-proteome and are indicated by red arrows.

Although the high overlap observed between sulfoxidation and the other PTMs may suggest a functional coupling of these covalent reversible modifications, further analyses designed to exclude sampling biases are needed before reaching such a conclusion. Indeed, protein abundance is a major factor for the detection of PTMs by mass spectrometry. Thus, avoiding abundance bias in the functional annotation of post-translationally modified proteins is a real concern^28^. To account for potential biases due to differential protein abundance, the Jurkat cell proteome was randomly sampled so that the protein abundance distribution within each random sample and the protein abundance distribution of the sulfoxide proteome were indistinguishable (Fig. 2A). Using these abundance-corrected random samples, we generated empirically new null distributions that allowed us to conclude that protein-bound methionine oxidation is linked to phosphorylation, acetylation and ubiquitination (Fig. 2B–D, respectively) in a manner that is unrelated to protein abundance.

### In the primary protein structure, MetO and phosphosites are closer than expected by chance

**Figure 3:**
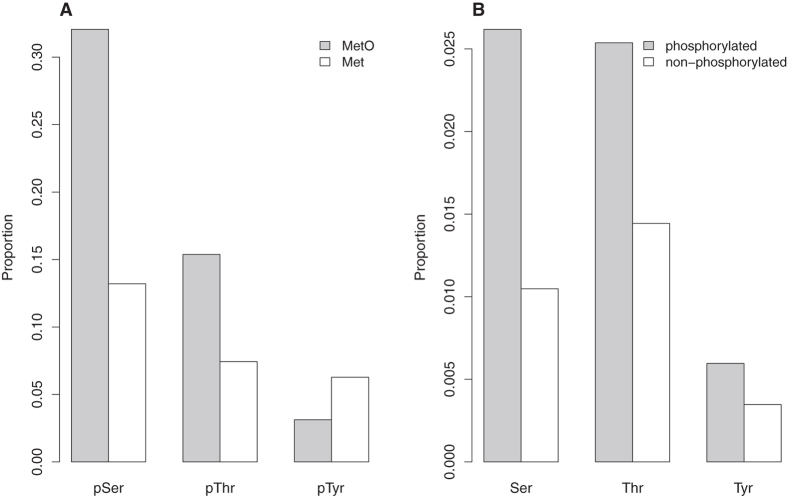
Proximity between MetO and phosphosites. For each methyonyl residue (either non-oxidized, Met, or oxidized methionine, MetO), the distances on the primary structure to the closest phosphosite and to the closest non-phosphorylatable phospho-acceptor were computed. (**A**) The proportion of MetO (grey bars) and Met (white bars) located at less than 7 residues from a phosphosite is plotted against the specific phosphosite. (**B**) The proportion of MetO closer than 7 residues from either a phosphosite (grey bars) or a non-phosphorylatable phosphoacceptor (white bars) is shown. All the compared proportions were significantly different (p-value < 10^−5^, non-parametric test for two proportions using Yates’ correction for continuity).

From the 774 proteins containing methionine sulfoxide, 758 are also phosphoproteins. These 758 proteins account for 13,880 methyonyl residues, for which 927 are detected as MetO after oxidative stress. We next asked whether those methionine residues that are oxidized *in vivo* under stress conditions (MetO) tend to be located closer to phosphosites when compared to methionine residues that are not oxidized (Met) within the same protein. To this end, the distance in the primary structure between each methionine and its closest phosphosite was computed. The mean distances for MetO and Met were 23 and 42, respectively (p-value < 2.2 10^−16^, Welch’s t-test). Nevertheless, since most phosphorylation motifs are less than a dozen residues in length^29^ (six residues from the phosphosite), we were interested in comparing the lower tails of the distributions rather than the means. Therefore, the proportions of MetO and Met that were found less than 7 residues away from any phosphosite were computed (0.41 and 0.22, respectively) and compared (p-value < 2.2 10^−16^, Fisher’s exact test). When this analysis was performed again and different types of phosphosites were distinguished, i.e., pSer, pThr and pTyr, it became clear that MetO sites are preferentially found around Ser/Thr phosphosites, while the behaviour was the opposite when the phosphosite considered was pTyr (Fig. 3A). Hitherto, we have compared MetO and Met with respect to their proximity to phosphosites. An alternative, and somehow complementary approach, is to focus on MetO and compare their distances to phosphosites (pSer, pThr and pTyr) with their distances to phospho-acceptors that are not phosphorylated (Ser, Thr and Tyr). Although these analyses may seem redundant, they are not. For instance, in a previous work, we reported that sequences surrounding MetO do not exhibit a preference for neighbouring tyrosine residues^30^, which is in line with our observation that there is only a small proportion of MetO near pTyr when compared to Met. This observation confirms and extends our previous results, since we can now affirm that MetO, in addition to disliking Tyr, also seems to avoid pTyr (Fig. 3A). However, when we focused the analysis on the few MetO that are close to tyrosines, we found that the tyrosines near MetO are preferentially phosphorylated (Fig. 3B). In other words, although MetO may avoid both Tyr and p-Tyr, the analysis summarized in Fig. 3B allows us to conclude that non-phosphorylatable tyrosines are more effectively excluded from the MetO environment.

### The proximity between sulfoxidation and phosphorylation sites is not accounted for by their co-localization within unstructured PTM clusters

**Figure 4:**
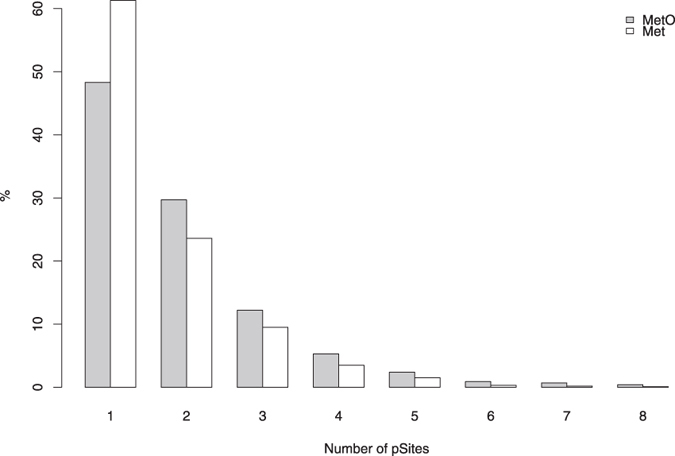
Distribution of the number of phosphosites in the environment of Met (white bars) and MetO (grey bars) residues. The number of phosphosites within a 10 residue window centred at the methionine (either oxidized or non-oxidized) was computed. The distribution of this variable is shown.

Phosphosites are often located in clusters found within unstructured regions of the protein exposed to the solvent^31^. Because solvent accessibility is an important determinant of methionine oxidation^30^, we wondered whether the proximity of sulfoxidation and phosphorylation sites described above may be accounted for by the presence of MetO within these clusters of phosphosites. Therefore, we next investigated whether MetO are preferentially positioned along these clusters. For this purpose, we focused on those methionine residues, regardless of their oxidation status, that contained at least one phosphosite within a 10 residue radius interval, centred at the methionine under analysis. Although methionine sulfoxides tend to concentrate a higher number of phosphosites into their surroundings when compared to non-oxidized methionines (Fig. 4), we could not conclude that MetO are preferably found within phosphosite clusters, as the percentage of MetO being accompanied by more than 3 phosphosites was only 10%. Nevertheless, there exist proteins such as ubiquitin-associated protein 2-like (Q14157) and kinesin light chain 2 (Q9H0B6) which possess sulfoxidable methionines (M466 and M612, respectively) that are surrounded by up to 8 different phosphosites.

**Figure 5:**
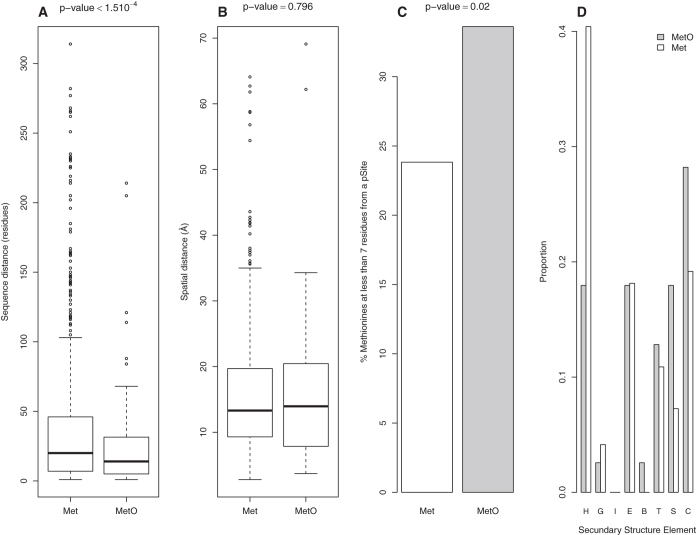
Proximity between MetO and phosphosites within structured proteins. For each methyonyl residue, regardless of its oxidation status, the distances on the primary structure as well as the spatial distance in Angstroms to the closest phosphosite were computed. (**A**) Comparison between Met and MetO with regard to their sequential distances to the nearest phosphosite (p-value = 1.5 10^−4^, Welch’s t-test). (**B**) Comparison between Met and MetO with regard to their spatial distances to the closest phosphosite (p-value = 0.796, Welch’s t-test). (**C**) The proportion of MetO (grey bars) and Met (white bars) located at less than 7 residues from a phosphosite is plotted (p-value = 0.02, Non-parametric 2-sample test for equality of proportions). (**D**) Plot of the proportions of MetO (grey bars) and Met (white bars) found in different secondary structure elements. H: α-helix; G: 3_10_-helix; I: π-helix; E: β-sheet; B: β-bridge; T: turn; S: bend; C: coil.

To further strengthen the conclusion that the closeness between MetO and pSites is not linked to their co-localization within unstructured regions, we compiled and analysed a dataset of 112 sulfoxidized phosphoproteins of known structure, which accounted for 855 and 124 non-oxidized and oxidized methionines, respectively. Both the distance in the primary structure and the spatial distance from each methionine to the closest phosphosite were recorded (Fig. 5). Although no differences in the spatial distance to the closest phosphosite were found between Met and MetO, the distance in residues was significantly lower for MetO when compared to Met (means = 24 and 37, respectively; p-value = 1.5 10^−4^, Welch’s t-test). These mean values, obtained using a small sample of structured proteins, are comparable with those reported above for the whole MetO-proteome. Furthermore, the proportion of MetO in structured proteins located at less than 7 residues from a phosphosite was significantly higher (p-value = 0.02, non-parametric 2-sample test for equality of proportions) with respect to their non-oxidized counterparts (Fig. 5C).

### Abundance of Met and MetO within phosphorylation site motifs

**Figure 6:**
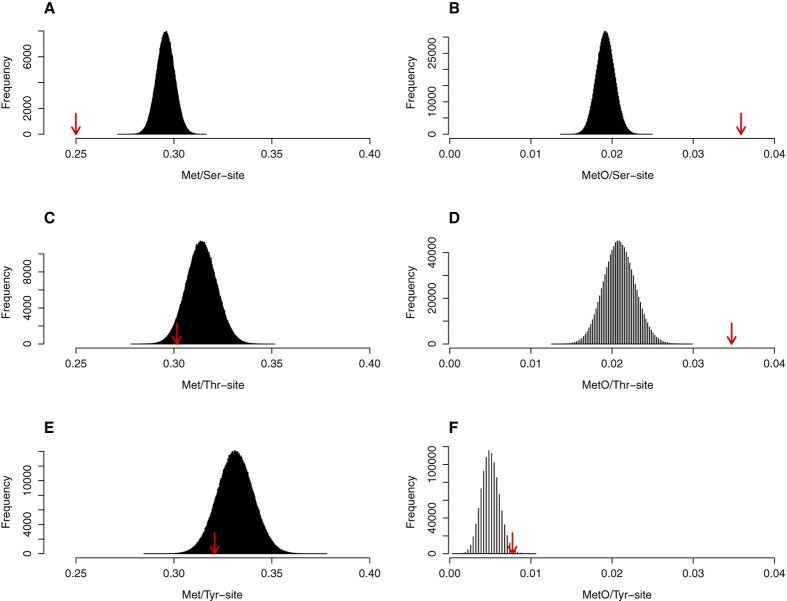
The low abundance of Met in phosphoserine motifs contrasts with their high content of MetO. The numbers of Met (regardless of its oxidation status) and MetO in a window of ±7 residues centred at the indicated phosphosites (pSer: top plots, pThr: middle plots and pTyr: bottom plots) were computed. The mean values of Met/pSite (left column plots) and MetO/pSite (right column plots) are indicated by an arrow. The empirical distributions were built by computing the mean for the numbers of Met and MetO per site in random samples taken from the phospho-acceptor population, regardless of their phosphorylation states (see Experimental Procedures for details). (**A**) The mean number of Met per pSer-site was 0.250 (arrow), which was significantly lower than the expected value for the mean number of Met per Ser-site, p-value < 10^−6^. (**B**) In contrast, the mean number of MetO per pSer-site, 0.036 (arrow), was significantly higher with respect to the expected mean number of MetO per Ser-site, p-value < 10^−6^. (**C**) The mean number of Met per pThr-site, 0.302 (arrow), although lower than the mean of the empirical distribution, did not reach statistical significance, p-value = 0.059. (**D**) However, the mean MetO/pThr-site, 0.035, was significantly higher, p-value < 10^−6^. (**E**) Mean Met/pTyr-site = 0.321, p-value = 0.127, (**F**) mean MetO/pTyr-site = 0.007, p-value = 0.013.

Methionine is a relatively rare amino acid, accounting for approximately 2.3% of all residues in human proteins^32^. However, the abundance of this rather non-polar amino acid is subject to both inter- and intramolecular variations. Thus, using our set of sulfoxidized proteins, we wanted to investigate potential differences in the frequencies of methionine around the three phospho-acceptors and whether these frequencies are related to the quality of being phosphorylatable. To this end, the variable Met/site (methionines per site) was defined as the number of methionines within a window of ±7 residues around the phospho-acceptor being analysed. Our results revealed notable differences in the distribution of Met and MetO around the different phospho-acceptors as well as around the different phosphosites (Fig. 6). Methionine is more abundant around tyrosine (mean Met/Tyr-site = 0.331, Fig. 6E), while serine is a phospho-acceptor with less methionine in its neighbourhood (mean Met/Ser-site = 0.295, Fig. 6A), and threonine is in between (mean Met/Thr-site = 0.314, Fig. 6C). When this analysis was restricted to phosphosites (phospho-acceptors that are phosphorylatable), phosphoserines stood out as methionine-averse sites (mean Met/pSer-site = 0.250, p-value < 10^−6^ with respect to mean Met/Ser-site, Fig. 6A). Surprisingly, despite this low frequency of methionine residues in the environment of pSer, the highest number of MetO was found around phosphoserines (mean MetO/pSer-site = 0.036, p-value < 10^−6^ with respect to mean MetO/Ser-site, Fig. 6B). By contrast, the average number of MetO around pTyr was only 0.007, in spite of tyrosine being the phosphoacceptor with the greatest abundance of methionine in its surroundings. Overall, these results strongly suggest that the oxidation of methionine residues within pSer motifs is a rather specific process.

**Figure 7:**
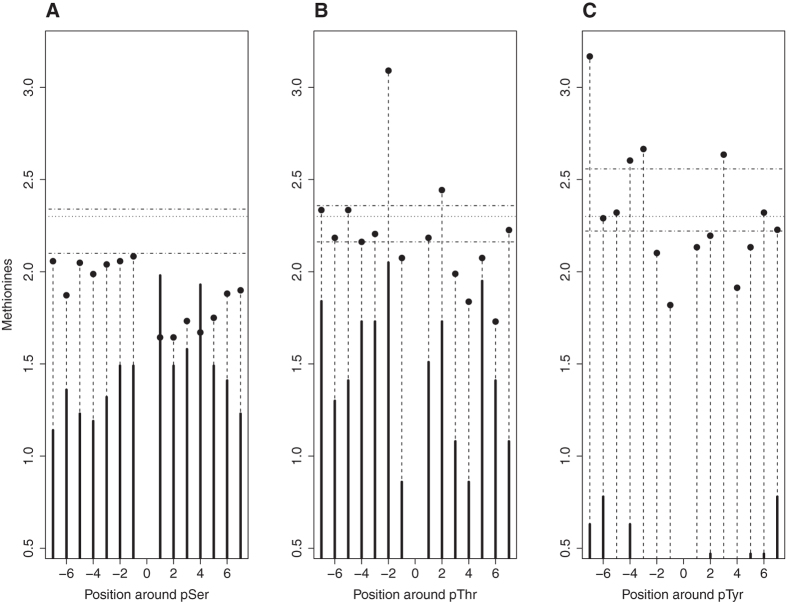
Positional distribution of Met and MetO around phosphosites. Around each phosphosite, 7 positions amino-terminal to the phosphosite (P-7 to P-1) and 7 positions carboxy-terminal to the phosphosite (P+1 to P+7) were considered. The frequencies of methionine (as percentages) at each of these 14 positions are represented as filled circles. Methionine frequencies were also computed around non-phosphorylatable phospho-acceptors. The horizontal dashed lines indicate the (mean – standard deviation) and the (mean + standard deviation) of these frequencies for non-phosphorylatable phospho-acceptor controls. The horizontal pointed line, used as a reference, indicates the frequency of methionine in the whole human proteome. The black bars represent the number of MetO counted every 500 phosphosites at the indicated position. The phosphosites analysed were pSer (**A**), pThr (**B**) and pTyr (**C**).

To further investigate this possibility, we next addressed the positional distribution of Met and MetO within the 14 residues surrounding pSer sites, as well as the other two types of phosphosites (Fig. 7). In agreement with the results presented above, pSer was the only type of phosphosite for which the frequency of methionine at any of the 14 considered positions was well below the frequencies observed at those positions around a non-phosphorylatable phospho-acceptor. Remarkably, methionine seems to be particularly avoided at positions carboxy-terminal to the phosphorylated serine (Fig. 7A, filled circles), despite the fact that it is precisely in these positions where we found the greatest number of oxidized methionines (Fig. 7A, black bars), again stressing the idea that the oxidation of methionine at those positions may be a specific process. This behaviour was particularly relevant at position P+1 and P+4 C-terminal to the pSer, where the lowest methionine frequencies coincided with the greatest abundance of MetO.

The importance of methionine oxidation at P+1 and P+4 positions was further reinforced by the results obtained using a different methodological approach. To this respect, we subjected a collection of 395 peptides known to exhibit both PTMs (phosphorylation and sulfoxidation) to the *motif-x* algorithm^33^,^34^. This program extracted only two motifs (SXXXM and SM) that were overrepresented among the peptides with multiple PTMs. The extracted motifs were defined by the existence of a methionyl residues either at position P+4 or at position P+1 C-terminal to the serine phospho-acceptor, respectively (see Fig. S1 from supplementary material). In contrast, *motif-x* fails to detect any motif in the control group formed by peptides containing both serine and methionine within a 15-residue window, which have not been reported to be target of multiple PTMs.

### Met/MetO as specificity determinants of Ser/Thr protein kinases

**Figure 8:**
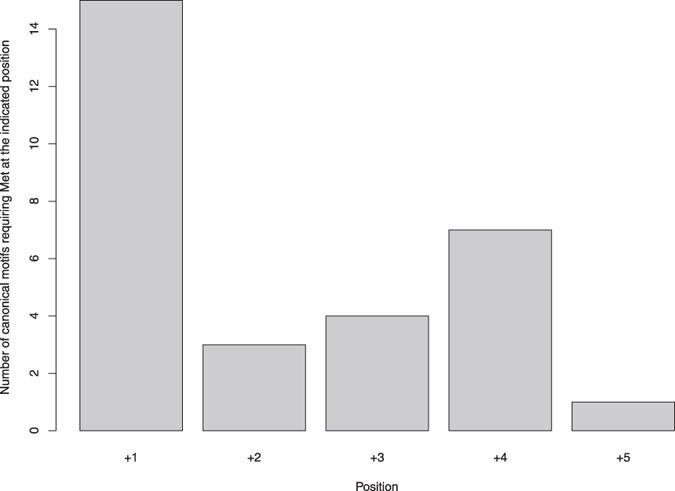
Positions at which methionine is found as being determinant within recognition motifs. From the literature, we gathered 33 canonical recognition motifs of Ser/Thr protein kinases involving methionine. Among the positions to the C-terminal side of pSer, as shown by the histogram, P+1 and P+4 were the positions in which methionine appeared more often as a specificity determinant.

A large number of studies on a wide variety of protein kinases have led to the conclusion that the selection of target substrates by protein kinases is strongly influenced by the amino acid sequence surrounding the phospho-acceptor site^29^. The amino acids within these environments that either promote or compromise phosphorylation are referred to as specificity determinants^35^,^36^. In the context of the current work, we focused our attention on the motifs that involve methionine as a determinant. Thus, we gleaned from the literature the Ser/Thr protein kinase substrate motifs containing methionine. We were able to identify 33 canonical recognition motifs in which methionine may play a role as a specificity determinant, accounting for approximately 20 different Ser/Thr protein kinases (Table S1). When we analysed the positions at which methionine appeared as a determinant, the results revealed P+1 and P+4 as relevant positions (Fig. 8).

**Figure 9:**
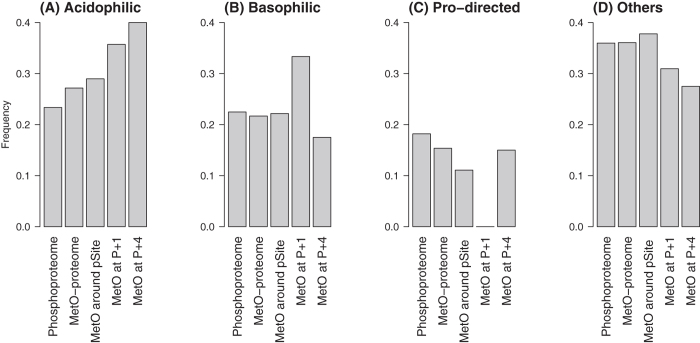
Classification of the phosphoserine sites from different subsets of the human proteome. Each phosphoserine site was assigned to one of the most general kinase recognition sequence categories: (**A**) acidic, (**B**) basic, (**C**) Pro-directed, and (**E**) “others”. The frequencies of each category within different subsets are shown. The considered subsets are: the whole phosphoproteome, the MetO-proteome, pSer containing MetO within a ±7 residues window centred at the phosphosite, pSer containing MetO at P+1 and pSer containing MetO at P+4.

According to the physicochemical properties of the residues present in the kinase substrates that are used as determinants for site recognition, a rough classification of Ser/Thr protein kinases into three main groups can be established: Pro-directed, basophilic and acidophilic^35^. To better understand the classes of kinases involved in the positional effects described above, we organized the phosphoserine sites from the human phosphoproteome into these three general sequence categories. Afterwards, we compared the frequencies of each category within different subsets of phosphosites: (i) the whole human phosphoproteome, (ii) pSer from the MetO-proteome, (iii) pSer containing MetO within a ±7 residue window centred at the pSite, (iv) pSer containing MetO at position P+1 and (v) pSer containing MetO at position P+4. The results of this analysis unveiled a link between acidic motifs and the presence of MetO (Fig. 9A). We also observed an enrichment of basophilic motifs among kinase substrates presenting MetO at P+1 (Fig. 9B), which takes place at the expense of a decrease in Pro-directed motifs (Fig. 9C).

### GO enrichment analysis of phosphoproteins containing MetO at P+1 and/or P+4

**Figure 10:**
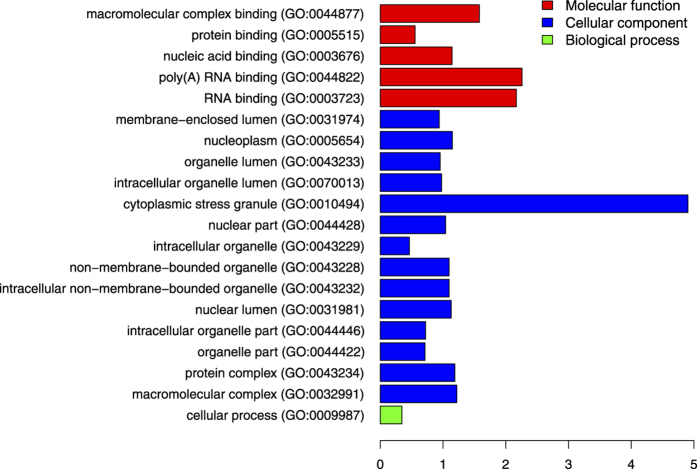
GO term enrichment analysis. The test set formed by human phosphoserine proteins containing MetO at P+1 and/or P+4 is enriched (p-value < 0.05, hypergeometric test with Bonferroni correction) for the GO terms indicated in the vertical axis. The abscissa axis shows the log_2_ fold-enrichment. Fold-enrichment is defined as the ratio of the number of proteins annotated with the GO term in the test set to the number of proteins annotated with such term in the background set (human phosphoproteome).

**Figure 11:**
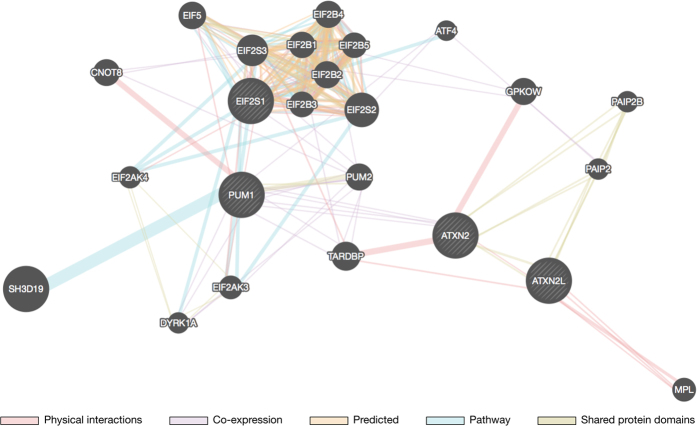
Motif network analysis of phosphoserine proteins containing MetO at P+1 and/or P+4. Four genes corresponding to sulfoxidized phosphoproteins found in stress granules (black nodes with white strips) were uploaded to GeneMANIA to produce an interaction network with predicted function. Non-striped nodes represent 20 genes predicted to be related to the four genes that were uploaded.

The results presented above suggest that the sulfoxidation of methionine at either position P+1 or P+4 within the substrate recognition motif may fulfil a regulatory function in transmitting information via phosphorylation. Thus, we conducted GO enrichment analysis of the sequences containing MetO at any of these two positions. The analysis was performed versus a reference list formed by all the human phosphoproteins. The result is shown in Fig. 10 and highlights a set of GO terms related to the control of gene expression that were significantly enriched (p-value < 0.05, hypergeometric test) in our dataset. The highest enrichment (approximately 30-fold) was observed for proteins annotated with the term “cytoplasmic stress granule” (Fig. 10). Stress granules are discrete cytoplasmic inclusions into which stalled translation initiation complexes are dynamically recruited in cells subjected to a variety of environmental stresses^37^. Table 1 provides details regarding the sulfoxidized phosphoproteins linked to stress granules. These four proteins are related in a well-defined single protein network (Fig. 11), and according to the GO enrichment analysis the functions predicted by GeneMANIA included “cytoplasmic stress granule”, “regulation of translational initiation” and “response to heat”.

## Discussion

While the regulatory importance of protein phosphorylation has long been known, the view that multiple and interacting PTMs represent a further gain in regulatory capabilities has only been more recently appreciated^38^. Although methionine sulfoxidation is generally perceived as an inevitable damage derived from oxidative stress, the notion of reversible methionine oxidation as a functional regulatory post-translational modification is gaining experimental support^39^,^40^,^41^. In addition to the direct effect on protein function, methionine sulfoxidation has been recently postulated as a mechanism to couple oxidative signals to changes in protein phosphorylation. This hypothesis derives from studies on two unrelated plant enzymes, mitochondrial pyruvate dehydrogenase^20^ and cytoplasmic nitrate reductase^19^, both of which have a methionine residue proximal to a regulatory phosphorylation site. In both cases, the authors demonstrated that the oxidation of these methionine residues resulted in the inhibition of phosphorylation, suggesting that the oxidation status of these methionines allows the enzymes to monitor oxidative stress and subsequently code this information in terms of phosphorylation patterns. In this way, crosstalk between these two PTMs may serve to fine-tune the cellular response to oxidative signals. Although appealing, this hypothesis is currently based on the study of only two proteins. If these observations prove to be valid in a more general context, it would further support a relevant role for methionine oxidation in the regulation of protein function. Therefore, the current study was performed to evaluate the potential of such proteome-wide crosstalk. However, the coexistence of multiple PTMs on the same protein is virtually impossible to determine from bottom-up proteomic analyses, while top-down approaches to this task are extraordinarily challenging in complex samples^42^; no attempt has been made until now to address the coexistence of phosphorylation and sulfoxidation sites on the same protein. Here, we have instead made use of a computational approach employing a previously described large-scale quantitative sulfoxidation data set^23^ which was crossed with data from the known human phosphoproteome^43^. In contrast to the phosphoproteomic data, which were derived from a vast number of different studies, the MetO-proteome data come from a single study using Jurkat cells subjected to oxidative stress. Thus, any future effort to expand on the current knowledge about the MetO-proteome (different tissues, species and experimental conditions) will be very valuable.

On the other hand, since the proteomic data we used in the current study were derived from bottom-up approaches, there is a caveat. Both the conversion of proteins into peptides in these so-called bottom-up approaches^44^ and the posterior peptide enrichment for detecting PTMs^45^ are methodological procedures liable to introduce sequence biases among the detected peptides. Therefore, the possibility, although extremely unlikely, is that the relationship of proximity we have described for MetO and pSer may be partially influenced by such biases.

Still, an immediate conclusion derived from our approach is that the set of proteins containing MetO is significantly enriched in phosphoproteins. In fact, as many as 98% of the proteins being sulfoxidized are also phosphorylatable. To contrast the statistical significance of such a high overlap, we built a null distribution by randomly sampling the human proteome. In such a way, the proportion of all cellular proteins that were phosphorylatable was estimated to be 83%, significantly lower than that observed among proteins from the MetO-proteome (p-value < 10^−6^). Our estimate of the proportion of human proteins containing covalently bound phosphate may seem high when compared to the 30–40% commonly cited estimates^26^,^46^. However, it should be noted that these estimates were based on *in situ* radiolabelling and 2D electrophoresis^35^, while more recent calculations based on high-throughput technologies suggest that at least three-quarters of the expressed proteome can be phosphorylated^47^. In any event, it seems obvious that the MetO-proteome exhibits a remarkable enrichment of phosphoproteins.

This observation, although encouraging, is by itself far from sufficient to conclude that crosstalk between phosphorylation and methionine sulfoxidation broadly occurs. Hence, with the aim of gathering evidence supporting such a role for methionine sulfoxidation, we reasoned that a testable hypothesis could be posed. For instance, if reversible oxidation of methionine has any influence on the ability of a nearby phospho-acceptor to be phosphorylated, oxidation sites should be selectively targeted rather than randomly occurring. Our results strongly suggest that this was the case. Thus, while phosphosite motifs proved to be methionine-poor regions, they harboured more MetO than their non-phosphorylatable counterparts (Fig. 6). This finding, which is contrary to the mass action effect, suggests a high degree of selectivity in the oxidation of methionine residues located in the proximities of phosphorylation sites. This effect was particularly remarkable for motifs with methionine at positions P+1 or P+4 (Figs 7 and 8), which are known to harbour hydrophobic residues that serve as recognition elements for some protein kinases (Table S1). Furthermore, the relevance of methionine at these positions was further stressed by the results obtained using *motif-x* (Fig. S1). For those proteins in which the required hydrophobic residue is methionine, its oxidation to MetO would interfere with the recognition process because the oxidation reaction converts the side chain of this amino acid from hydrophobic to polar^48^. This scenario would be congruent with a regulatory (inhibitory) role for the reversible oxidation of methionine at these specific positions.

For the reasons discussed above, we focused our attention on positions P+1 and P+4 from phosphoserine motifs. However, it is worth mentioning that methionine also appears as a specificity determinant at other positions. In this regard, some protein kinases such as calmodulin-dependent protein kinase II (CamKII), strongly select for glutamine at position P-2 and also exhibit a great tolerance for methionine at this position^49^. Interestingly, in mutagenesis experiments, glutamine is the amino acid of choice to mimic the properties of methionine sulfoxide^50^,^51^,^52^. Indeed, both glutamine and MetO present an oxygen atom at the same position in their side chains and exhibit roughly the same hydrophobicity index value^48^. Hence, it is tempting to speculate that the oxidation of methionyl residues from substrates of this group of protein kinases, which in addition to CamKII also include JNK1, p38a MAPK and PKD (see Table S1), may represent a signal that promotes the phosphorylation of these substrates. Furthermore, such a mechanism would provide a rationale for the well-known involvement of these kinases as mediators of oxidative stress^53^,^54^,^55^,^56^.

Our results also revealed that phosphoserine motifs containing MetO may be preferentially phosphorylated by acidophilic protein kinases (Fig. 9). The biological significance of such a finding, if any, is unknown. However, this observation is in agreement with previous studies reporting an abundance of acidic residues around MetO^30^,^57^. The results presented herein suggest that the previously observed propensity of MetO and glutamate to colocalize may be related to the crosstalk between sulfoxidation and acidophilic protein kinases, although such a causal relationship remains to be proven.

To gain further insight into the processes that may be regulated by crosstalk between sulfoxidation and phosphorylation, we conducted GO analyses. The results indicated that the occurrence of MetO near phosphoserine sites was more prevalent in proteins related to the control of translation and stress related proteins (Fig. 10). Of particular note was the 30-fold enrichment in proteins related to cytoplasmic stress granules (SGs). Cytoplasmic SGs are multimolecular aggregates of stalled translation pre-initiation complexes that prevent the accumulation of misfolded proteins and protect RNAs from harmful conditions. They are formed in response to diverse types of stress and contribute to cell survival not only by suppressing translation but also by sequestering apoptotic factors. Global inhibition of protein synthesis is a common response to stress conditions. In fact, translational control is a key component of the cellular response to oxidative stress. Indeed, oxidative stress induced by exposure to H_2_O_2_ elicits a complex translational reprogramming that is fundamental for adaptation to the stress^58^. It was shown that hydrogen peroxide causes an inhibition of translation initiation dependent on the phosphorylation of the α-subunit of eukaryotic initiation factor-2 (eIF2α). Phosphorylation of eIF2α is an early event in the assembly of SGs that serves as an important checkpoint under which general protein synthesis is blocked, thus allowing cells to either recuperate from stress or be eliminated if the damage is beyond repair^59^. In the current study, we have identified eIF2α, together with other proteins related to SGs (Table 1), as a potential target for crosstalk between sulfoxidation and phosphorylation. The functional relationship of these four proteins was further supported by a network analysis (Fig. 11). Met 223 of eIF2α has been shown to be extensively oxidized to MetO in cells that were treated with H_2_O_2_^23^. On the other hand, this sulfoxidation site is four residues away from a phosphorylatable serine residue belonging to an acidophilic motif. The identification of these candidate proteins, where methionine sulfoxidation may be involved in coupling oxidative signals with phosphorylation, should stimulate further research on PTM crosstalk during the cellular response to oxidative stress.

## Concluding remarks

Protein phosphorylation is a key event in the cellular response to oxidative stress. Despite the enormous research efforts that has been devoted to the study of protein phosphorylation, the molecular mechanisms coupling oxidative signals to changes in phosphorylation remain poorly understood. It has been proposed that oxidation of methionine to MetO, a reaction that converts the side chain of this amino acid from hydrophobic to hydrophilic, may provide the basis for regulating the specificity of protein kinase-substrate interactions. Statistically rigorous analyses of proteome data support this hypothesis. Thus, our results clearly demonstrate that (i) nearly all of the proteins that are sulfoxidized after exposure to H_2_O_2_ are also phosphoproteins, (ii) MetO and pSites are spatially related in the primary structure of protein kinase substrates, (iii) oxidation of methionine harboured within phosphorylation motifs is a highly selective process and (iv) the described relationship between sulfoxidation and phosphorylation is more prevalent among stress-related proteins. Overall, we propose that phosphorylation and methionine sulfoxidation crosstalk broadly occurs. Furthermore, our results should stimulate further investigation to determine the role of the interplay between these two PTMs in the formation and dynamics of stress granules after an oxidative challenge.

## Methods

### Data collection

Data regarding protein-bound methionines that were oxidized in Jurkat cells subjected to H_2_O_2_ stress were taken from Table S1 in the supplementary material of a previous work^23^. These data were further manually curated to exclude ambiguous entries as well as those methionyl residues that did not show an extensive oxidation (below 20% oxidation). Finally, a total of 774 different sulfoxidized proteins were included in the dataset referred to as the MetO-proteome. Human phosphorylated proteins (19,238), ubiquitinated proteins (8,964) and acetylated proteins (6,965), as well as their corresponding sites, were retrieved from PhosphoSite^43^.

### Empirical null distribution for the co-occurrence of PTMs

To evaluate the statistical significance of the observed high overlap between methionine sulfoxidation and other PTMs, such as phosphorylation, acetylation and ubiquitination, empirical null distributions were generated as described. One million samples, each including 774 human proteins (the size of our MetO-proteome dataset), were randomly collected from UniProt. For each sample, the proportions of phosphorylatable, ubiquitinable and acetylable proteins were computed and used to build null distributions. We also generated empirical null distributions corrected for protein abundance. For this purpose, we collected abundance data for proteins expressed in Jurkat cells^60^. The MetO-proteome dataset was restricted to 533 proteins for which we had data regarding their abundances. Then, one million random samples, each including 533 proteins, were collected from the Jurkat cell proteome. Although this sampling was random, we constrained it to provide samples showing a protein abundance distribution that was indistinguishable from the MetO-proteome. To this end, a quarter of the sample was randomly collected among all the proteins from the Jurkat cell proteome showing abundances within the first quartile of the sulfoxide proteome abundance distribution. Another quarter was sampled among the second quartile, and so on. Further methodological details, raw data and a script implementing this analysis can be downloaded from https://github.com/jcaledo/MetO_pSTY.

### Empirical null distributions for the concentration of Met and MetO around phosphoacceptors

We identified 758 proteins from the MetO-proteome as phosphoproteins. These 758 proteins accounted for 44,337 serines, 29,434 threonines and 13,601 tyrosines. Herein, and throughout the current paper, we shall use the term phospho-acceptor to refer to any of these 87,372 residues, regardless of their ability to be phosphorylated. From this set of phospho-acceptors, 11,062, 4,550 and 3,105 have been described as phosphorylatable serines, threonines and tyrosines, respectively. For each of these phosphosites, the number of total methionines (Met + MetO) and oxidized methionines (MetO) within a window of ±7 residues centred at the phospho-acceptor was computed. In this way, the mean values for the total Met per phosphosite and MetO per phosphosite were calculated. To contrast these mean values, null distributions were generated empirically as described below. For instance, to contrast the mean number of Met per pSer site, 10^6^ random samples of size 11,062 were taken from the set of 44,337 serine phospho-acceptor. After computing the mean number of Met (respectively MetO) per serine site in each of these samples, we obtained a collection of 10^6^ mean values that were used as the null distribution. Similar procedures were applied for pThr and pTyr sites.

### Classification of phosphoserine motifs

The Ser/Thr protein kinases fall into three major subgroups: Pro-directed, basophilic and acidophilic, on the basis of the types of preferred substrate sequences^35^. All of the phosphoserine sites from the human phosphoproteome were organized into these three general sequence categories using a binary decision tree as described elsewhere^61^. Briefly, if proline is found at P+1 (position 1 C-terminal to the pSer), then the site is assigned to the class “Pro-directed”; if 5 or more aspartates or glutamates are found between P+1 and P+6, then the site is classified as “Acidophilic”; if arginine or lysine is found at P-3 (position 3 N-terminal to the pSer) then the site is considered to be “Basophilic”; when aspartate or glutamate is found at P+1 or P+2 or P+3, the site is assigned to the category “Acidophilic”; if 2 or more arginines or lysines are located between P-6 and P-1, the site is considered “Basophilic”; otherwise the pSer site is labelled as “Others”.

### Phosphoserine motif extraction

We started assembling a collection of 11,136 human peptide sequences centred at serine and containing at least one methionine within a window of ±7 residues. Raw data formatted as a R data frame named “Acceptor.rda” can be downloaded from https://github.com/jcaledo/MetO_pSTY. This collection was split into two complementary subsets. One, referred to as multiple PTMs set, was formed by 395 peptides where both the central serine and at least one methionine have been empirically detected as modified residues. The other subset, labelled as control, was formed by the remaining 10,741 peptides. To detect overrepresented motifs, we used the *motif-x* software v1.2 10.05.06^33^. Both, the multiple PTMs and the control data sets described above were used as foreground inputs, which were submitted as pre-aligned data sets to be analysed using the entire human proteome as background to empirically determine the conditional probabilities required for significance^33^. The significance (p-value threshold for the binomial probability) was set at 10^−35^ and the occurrence parameter was fixed at 10% of the total number of peptides in the analysed set.

### Secondary and tertiary structural data

A list of PDB identifiers for proteins belonging to the MetO-proteome was obtained using the PDB cross-reference from UniProt. After filtering out low-quality structures (for instance, those in which the target methionine and/or phospho-acceptor did not appear to be resolved), we assembled a collection of 112 unique proteins of known structures containing 979 methionines, 124 of which were oxidation-prone. For each methionine, the distance from the sulfur atom to the oxygen atom of the hydroxyl group from the closest phosphorylatable residue was computed with the aid of an *ad hoc* R script that relies on the package bio3d^62^. Secondary structure assignment was performed using DSSP^63^. The 3_10_, α and π helixes were grouped under the ‘helix’ category, β-sheet and β-bridge were considered as ‘strand’, while helix turn, bend and coil were grouped together as ‘coil’.

### Gene ontology enrichment and motif network analyses

Phosphoserine proteins containing MetO at P+1 and/or P+4 within its substrate recognition motif were subjected to GO term-enrichment analyses using the amiGO 2 web server^64^. The analyses were performed versus a reference list formed by all of the human phosphoproteins. A hypergeometric test with Bonferroni correction for multiple comparisons was used to select significantly (p-valued < 0.05) enriched terms. A gene interaction network was obtained for four sulfoxidized phosphoproteins found in stress granules using GeneMANIA^65^ with the automatically selected weighting method. GeneMANIA evaluates the false discovery in function prediction with the false discovery rate method.

## Additional Information

**How to cite this article:** Veredas, F. J. *et al*. Methionine residues around phosphorylation sites are preferentially oxidized *in vivo* under stress conditions. *Sci. Rep.* **7**, 40403; doi: 10.1038/srep40403 (2017).

**Publisher's note:** Springer Nature remains neutral with regard to jurisdictional claims in published maps and institutional affiliations.

## Supplementary Material

Supplementary Information

## Figures and Tables

**Table 1:** Sulfoxidized phosphoproteins found in stress granules.

Protein	Uniprot	Motif	pSer	MetO	Group
eIF2 subunit 1	P05198	LRAGLNC***S***TEN***M***PIK	219	223	Acidophilic
Pumilio homologue 1	Q14671	HAEHQVR***SM***DELNHD	124	125	Acidophilic
Ataxin-2	Q99700	QPSSTSE***SM***DQLLNK	814	815	Acidophilic
Ataxin-2-like protein	Q8WWM7	TKDKFTD***S***AIA***M***NSK	211	215	Basophilic

The columns pSer and MetO give the positions in the primary structure of the phosphorylation and sulfoxidation sites, respectively.
