# Supplementary material for: Methionine residues around phosphorylation sites are preferentially oxidized in vivo under stress conditions
Source: Sci Rep. 2017 Jan 12;7:40403. doi: 10.1038/srep40403 (PMC5227694; doi:10.1038/srep40403)
Supplement: Supplementary Information [file srep40403-s1.pdf]

Methionine residues around phosphorylation sites are preferentially oxidized *in vivo* under stress conditions

Francisco J. Veredas[1], Francisco R. Cantón[2], J. Carlos Aledo[2,*]

[1]Departamento de Lenguajes y Ciencias de la Computación, Universidad de Málaga. 29071-Málaga, Spain.

[2]Departamento de Biología Molecular y Bioquímica, Facultad de Ciencias. Universidad de Málaga. 29071-Málaga, Spain.

*Corresponding author: JCA, caledo@uma.es

Figure S1. Overrepresented motifs among peptides with multiple PTMs. Using the algorithm *motif-x*, two motifs (methionine at position P+4 and P+1) were identified as being significantly enriched among peptides known to show both types of PTMs: serine phosphorylation and methionine sulfoxidation. The control sample was formed by peptides containing both serine and methionine within a 15-residues window, which have not been shown to be target of multiple PTMs. No motif was found within the control group.

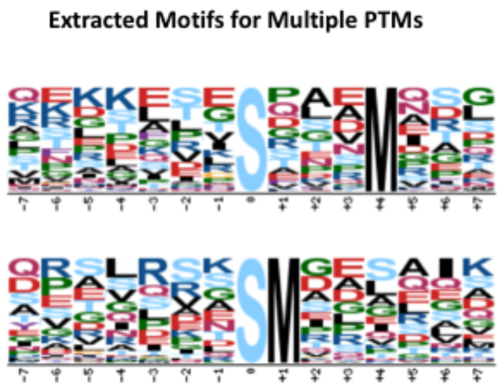

Table S1. Canonical recognition motifs from Ser/Thr protein kinase substrates containing methionine as specificity determinant. The first column (pink) lists the identified protein kinases. The second column (blue) shows the corresponding recognition motifs, with methionine in boldface. For each given motif, a link to the relevant publication can be found in the last column (green).

**Kinase**
Akt
AMP-activated protein kinase
AMP-activated protein kinase
AMP-activated protein kinase
ATM kinase
Aurora-A kinase
Calmodulin-dependent protein kinase I
Calmodulin-dependent protein kinase II
Calmodulin-dependent protein kinase II
Calmodulin-dependent protein kinase II
Calmodulin-dependent protein kinase IV
Casein Kinase I
Chk1
Doublecortin kinase-1
ERK1
ERK2
HMGCoA Reductase kinase
JNK1
MAPKAPK2
MEKK
NIMA kinase
NIMA kinase
p70 Ribosomal S6 kinase
Phosphorylase kinase
PKA
PKA
PKC beta
PKC mu
PKC theta
PKC theta
PKD
Plk1
TGF beta receptor kinase

Motif
[R/Q/K]-[R/K/N/Q/P/H]-[R/K]-[R/S/T]-[N/K/Q/H/D/P]-pS-[F/W/I/**M**/N/S]-[S/T/H]-[R/S/K]-[S/T/P/Q]
[**M**/V/L/I/F]-[R/K/H]-X-X-X-[pS/pT]-X-X-X-[**M**/V/L/I/F]
[**M**/V/L/I]-X-X-[R/K/H]-X-[pS/pT]-X-X-X-[**M**/V/L/I]
**[M**/V/L/I/F]-[R/K/H]-X-X-[pS/pT]-X-X-X-[**M**/V/L/I/F]
[P/L/I/**M**]-X-[L/I/D/E]-pS-Q
[R/K/N]-R-X-[pS/pT]-**[M**/L/V/I]
[**M**/V/L/I/F]-X-R-X-X-[pS/pT]-X-X-X-[**M**/V/L/I/F]
[**M**/I/L/V/F/Y]-X-R-X-X-[pS/pT]-[**M**/I/L/V/F/Y]
[K/F]-[R/K]-[Q/**M**]-[Q/**M**/K/L/F]-pS-[F/I/**M**/L/V]-[D/E/I]-[L/**M**/K/I]-[F/K]
[**M**/V/L/I/F]-X-[R/K]-X-X-[pS/pT]-X-X
[**M**/I/L/V/F/Y]-X-R-X-X-[pS/pT]
[pS/pT]-X-X-X-[S/T]-[**M**/L/V/I/F]
[**M**/I/L/V]-X-[R/K]-X-X-[pS/pT]
[I/L/V/F/**M**]-R-R-X-X-[pS/pT]-[I/L/**M**/V/F]
[T/P/S]-[G/P/E/Y]-[P/L/I]-[L/**M**/P]-pS-P-[G/P/F]-[P/F/G/Y]-[F/Y/I]
[D/Y/W/E]-[C]-[P/S/C/E]-[P/C/S/L/T/V]-[L/**M**/T]-pS-[P/A]-[T/S/G/R/C/F]-[W/P/S]-[W/F]
[**M**/L/V/I/F]-[R/K/H]-X-X-pS-X-X-X-[**M**/L/V/I/F]
G-P-[Q/**M**]-pS-P-I
[L/F/I]-X-X-X-R-[Q/S/T]-L-[pS/pT]-[**M**/L/I/V]
R-R-F-G-pS-[**M**/L/V/I/F]-R-R-[**M**/L/V/I/F]
[R/N]-[F/L/**M**]-[R/K]-[R/K]-pS-[R/I/V/**M**]-[R/I/M/V]-[**M**/I/F/V]-[I/F/**M**]
R-F-[R/K]-[R/K]-pS-[R/I]-[R/I]-**M**-I
[R/K]-X-R-X-X-[pS/pT]-[**M**/L/V/I]
[F/**M**/K]-[R/K]-[**M**/R/Q/F]-[**M**/F/L/I]-pS-[F/I/**M**/L]-[F/R/K]-[L/I]-[F/L/I]
R-R-X-pS-[**M**/I/L/V/F/Y]
[R/C/P/K]-[R/A/P]-[R/K]-[R/K/S]-[N/L/S/**M**/P]-pS-[I/L/V/C]-[S/P/H/Q]-[S/W/Q]-[S/L/G]
[L/R/F]-[R/K]-R-[K/Q]-G-pS-[F/**M**]-K-K-X-A
[L/V]-[V/L/A]-R-[Q/K/E]-**M**-pS
[R/F/W/**M**]-[W/A/K/S]-[R/S/K/H]-[R/H/S/Q]-[R/K/N/P/G/Q]-pS-[I/F/R/V/K/S/L/**M**]-[K/**M**/R/S/T]-[R/S/K/W]-[R/K/G]
F-X-R-X-X-pS-[F/**M**]-[F/**M**]
[L/V/I]-[R/K/Q]-[R/K]-[R/K/T/Q/**M**]-[N/K/R/L/**M**/H]-pS-[F/W/I/**M**/L/V]-[S/N]-[R/S/P/Y/W]-[S/R/N/L]
[D/E]-X-[pS/pT]-[I/L/V/**M**]-X-[D/E]
[R/K/Q/N]-[**M**/C/W]-[R/T/S/N]-[E/D/S/N]-[R/K/E/D/N]-pS-[S/D/E]-[S/G/C/D]-[S/**M**/R/N]-[N/H/S/R/C]

PubMed

http://www.ncbi.nlm.nih.gov/entrez/query.fcgi?cmd=Retrieve&db=pubmed&dopt=Abstract&list_uids=15782149
http://www.ncbi.nlm.nih.gov/entrez/query.fcgi?cmd=Retrieve&db=pubmed&dopt=Abstract&list_uids=7902296,7698321
http://www.ncbi.nlm.nih.gov/entrez/query.fcgi?cmd=Retrieve&db=pubmed&dopt=Abstract&list_uids=7902296
http://www.ncbi.nlm.nih.gov/entrez/query.fcgi?cmd=Retrieve&db=pubmed&dopt=Abstract&list_uids=7698321
http://www.ncbi.nlm.nih.gov/entrez/query.fcgi?cmd=Retrieve&db=pubmed&dopt=Abstract&list_uids=10608806
http://www.ncbi.nlm.nih.gov/entrez/query.fcgi?cmd=Retrieve&db=pubmed&dopt=Abstract&list_uids=16083426
http://www.ncbi.nlm.nih.gov/entrez/query.fcgi?cmd=Retrieve&db=pubmed&dopt=Abstract&list_uids=9452427,7698321,8022798
http://www.ncbi.nlm.nih.gov/entrez/query.fcgi?cmd=Retrieve&db=pubmed&dopt=Abstract&list_uids=9452427
http://www.ncbi.nlm.nih.gov/entrez/query.fcgi?cmd=Retrieve&db=pubmed&dopt=Abstract&list_uids=8887677
http://www.ncbi.nlm.nih.gov/entrez/query.fcgi?cmd=Retrieve&db=pubmed&dopt=Abstract&list_uids=8280084
http://www.ncbi.nlm.nih.gov/entrez/query.fcgi?cmd=Retrieve&db=pubmed&dopt=Abstract&list_uids=9452427
http://www.ncbi.nlm.nih.gov/entrez/query.fcgi?cmd=Retrieve&db=pubmed&dopt=Abstract&list_uids=12925738
http://www.ncbi.nlm.nih.gov/entrez/query.fcgi?cmd=Retrieve&db=pubmed&dopt=Abstract&list_uids=10648819
http://www.ncbi.nlm.nih.gov/entrez/query.fcgi?cmd=Retrieve&db=pubmed&dopt=Abstract&list_uids=12590608
http://www.ncbi.nlm.nih.gov/entrez/query.fcgi?cmd=Retrieve&db=pubmed&dopt=Abstract&list_uids=8887677
http://www.ncbi.nlm.nih.gov/entrez/query.fcgi?cmd=Retrieve&db=pubmed&dopt=Abstract&list_uids=15782149
http://www.ncbi.nlm.nih.gov/entrez/query.fcgi?cmd=Retrieve&db=pubmed&dopt=Abstract&list_uids=7698321
http://www.ncbi.nlm.nih.gov/entrez/query.fcgi?cmd=Retrieve&db=pubmed&dopt=Abstract&list_uids=15629715
http://www.ncbi.nlm.nih.gov/entrez/query.fcgi?cmd=Retrieve&db=pubmed&dopt=Abstract&list_uids=15807522
http://www.ncbi.nlm.nih.gov/entrez/query.fcgi?cmd=Retrieve&db=pubmed&dopt=Abstract&list_uids=7874496
http://www.ncbi.nlm.nih.gov/entrez/query.fcgi?cmd=Retrieve&db=pubmed&dopt=Abstract&list_uids=8887677,1416988
http://www.ncbi.nlm.nih.gov/entrez/query.fcgi?cmd=Retrieve&db=pubmed&dopt=Abstract&list_uids=8120013,8982275
http://www.ncbi.nlm.nih.gov/entrez/query.fcgi?cmd=Retrieve&db=pubmed&dopt=Abstract&list_uids=7498520,1737763
http://www.ncbi.nlm.nih.gov/entrez/query.fcgi?cmd=Retrieve&db=pubmed&dopt=Abstract&list_uids=8887677
http://www.ncbi.nlm.nih.gov/entrez/query.fcgi?cmd=Retrieve&db=pubmed&dopt=Abstract&list_uids=8463304,194899
http://www.ncbi.nlm.nih.gov/entrez/query.fcgi?cmd=Retrieve&db=pubmed&dopt=Abstract&list_uids=12566450,10574945
http://www.ncbi.nlm.nih.gov/entrez/query.fcgi?cmd=Retrieve&db=pubmed&dopt=Abstract&list_uids=12566450,10574945
http://www.ncbi.nlm.nih.gov/entrez/query.fcgi?cmd=Retrieve&db=pubmed&dopt=Abstract&list_uids=8995387
http://www.ncbi.nlm.nih.gov/entrez/query.fcgi?cmd=Retrieve&db=pubmed&dopt=Abstract&list_uids=15782149
http://www.ncbi.nlm.nih.gov/entrez/query.fcgi?cmd=Retrieve&db=pubmed&dopt=Abstract&list_uids=8995387
http://www.ncbi.nlm.nih.gov/entrez/query.fcgi?cmd=Retrieve&db=pubmed&dopt=Abstract&list_uids=15782149
http://www.ncbi.nlm.nih.gov/entrez/query.fcgi?cmd=Retrieve&db=pubmed&dopt=Abstract&list_uids=12738781
http://www.ncbi.nlm.nih.gov/entrez/query.fcgi?cmd=Retrieve&db=pubmed&dopt=Abstract&list_uids=15782149
